# PriBeL: A primary betel leaf dataset from field and controlled environment

**DOI:** 10.1016/j.dib.2025.111674

**Published:** 2025-05-19

**Authors:** Gauri Mane, Raghav Bhise, Rutuja Kadam, Gagandeep Kaur, Devika Verma, Rupali Chopade, Gitanjali Shinde, Ghanshyam G. Tejani, Seyed Jalaleddin Mousavirad

**Affiliations:** a Department of Computer Science Engineering – Artificial Intelligence and Machine Learning, Vishwakarma Institute of Information Technology, Pune, India; b Symbiosis Institute of Technology, Nagpur Campus, Symbiosis International (Deemed University), Pune, India; c Department of Computer Science Engineering, Vishwakarma Institute of Technology, Pune, India,; d School of Engineering and Technology, DES Pune University, Pune, India,; e Department of Research Analytics, Saveetha Dental College and Hospitals, Saveetha Institute of Medical and Technical Sciences, Saveetha University, Chennai, 600077 India; f Applied Science Research Center, Applied Science Private University, Amman, 11937, Jordan; g Department of Computer and Electrical Engineering, Mid Sweden University, Sundsvall, 851 70, Sweden

**Keywords:** Agriculture, Betel leaves, Primary dataset, On field, Controlled environment

## Abstract

Essentially, visual identification of plant health is vital for research in agriculture and medicinal plants for important crops, both in terms of economics and pharmacology, such as betel leaves. The strong integration of AI-based methods in precision agriculture and herbal medicine quality control makes these systems effective only when trained on well-structured, diversified datasets.The plant betel leaf (Piper betle) is cultivated throughout the world for its medicinal, cultural, and economic importance, but improper classification and quality assessment of this plant occur because of environmental conditions and variations in handling. To solve this problem, we hereby present the Betel Leaf Dataset, which is systematically curated, consisting of 1,800 high-resolution images (1080 × 1080 pixels) exhibiting the three different conditions of betel leaves: Healthy (Fresh), Diseased, and Dried. The dataset was collected from Veer, Taluka-Purandar, Pune, India, under both natural and controlled conditions so that different appearances could be ensured. Categories include images that have been taken under varied light, backgrounds, and orientations, which comprehensively can cover all real variations in betel leaves. Hence, this systematically collected, standardized, and accessible dataset can enhance agricultural research in leaf classification studies and quality assessment techniques to facilitate better documentation and understanding of betel leaf characteristics. This dataset can be utilized in machine learning applications for plant disease detection, precision agriculture, and automated quality control systems.

Specifications Table

**Table:** 

Subject	Computer Sciences
Specific subject area	Betel Leaf Dataset for identification, classification and severity estimation
Type of data	Image
Data collection	The dataset comprises images of betel leaves collected from farms in Maharashtra with the assistance of agronomists. The images were captured using a Samsung Galaxy S23 (Pro Camera) having the main camera of 50 MP, f/1.8. All images were standardized to 1080×1080 pixels and saved in JPEG format for consistency. The indoor collection is done in controlled lighting and environment, while field collection is done only during the daytime. A total of 1,800 images were collected, classified healthy as 669, dried as 622, and diseased as 509.
Data source location	At Veer, Taluka-Purandar, District-Pune, Maharashtra, India. Latitude :18.1507784, Longitude :74.0872852
Data accessibility	Repository name: Betel Leaf Dataset: A Primary Dataset From Field And Controlled Environment Data identification number: 10.17632/btdym2t6mt.1 Direct URL to data: https://data.mendeley.com/datasets/btdym2t6mt/1
Related research article	None

## 1 Value of the Data

• Betel leaves are highly grown and best known within South and Southeast Asia as piper betles due to their culinary, cultural, and medical uses. In India, the leaves are mostly grown due to warm humid conditions in states such as West Bengal, Assam, Odisha, Karnataka, Tamil Nadu, and Andhra Pradesh. The evergreen vine, which thrives in these conditions, climbs between 1 and 3 meters high while reaching maturity within 4 to 6 months.
• Apart from farming, the betel leaf is an important cultural and economic commodity in South and Southeast Asia; it is for religious rituals, traditional medicine, and chewing betel quid (Paan) as a social event. It is also an important herb in Ayurveda and local medicine with antibacterial, anti-inflammatory, and antiseptic properties. It directly supports thousands of farmers and greatly contributes to the agricultural economy. These include some region-specific varieties like Bangla, Desi, Kapoori and Meetha Paan, and they differ from each other in terms of taste, texture and medicinal properties.
• This structured and labeled image dataset can be useful for training and evaluating machine learning (ML) and artificial intelligence (AI) models focusing on plant health monitoring, leaf classification, and disease detection, severity estimation of diseases and fertilizer, pesticide recommendations. Furthermore, this dataset will facilitate the development of the mobile applications and embedded systems in smart farming and agritech solutions for real-time leaf quality analysis, precision agriculture, and digital diagnostics.
• The dataset consists of a structured collection of pictures taken under controlled and field conditions, which are further divided into three categories: healthy, diseased, and dried leaves. This classification makes qualitative assessment and documentation possible through a completely informed understanding of betel leaf variation.
• This dataset alongside performing a systematic subdivision of betel leaves into discrete classes gives pivotal information regarding post-harvest conditions, environmental effects on growth, and health of leaves. In doing so, it is relevant for research and agricultural work since it guarantees in-depth identification, sorting, and analysis.

## 2 Background

**Fig 1:**
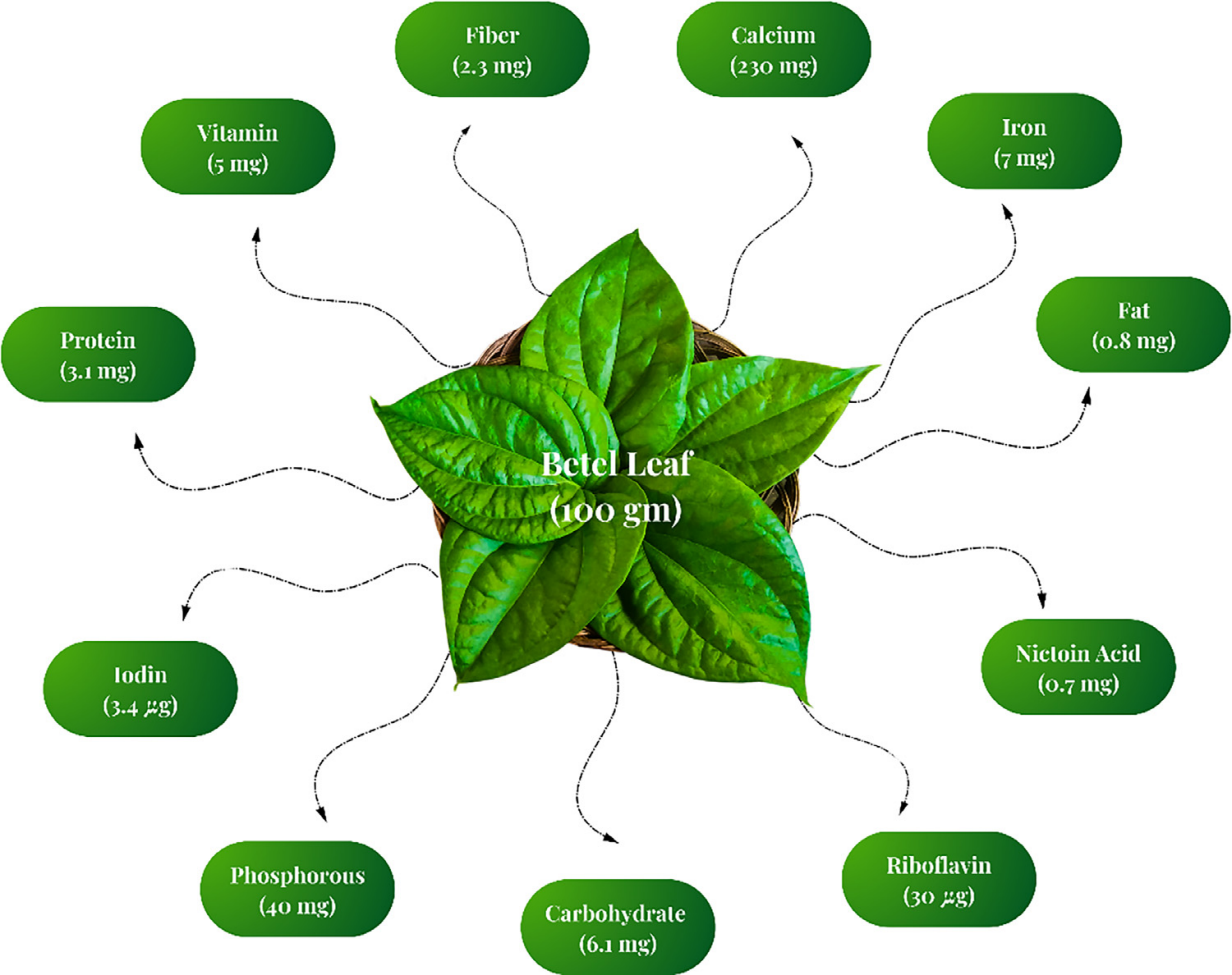
Constituents of betel leaf.

**Fig 2:**
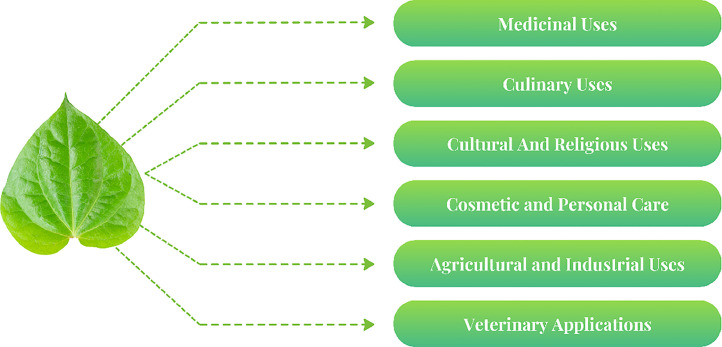
Applications of betel leaf.

Betel leaves belong to a nutritionally rich plant: they encompass essential macro- and micronutrients. A 100 g serving contains a rather good supply of calcium (230 mg), phosphorus (40 mg), and iron (7 mg); if this is not enough, this leaf provides nicotinic acid (0.7 mg), riboflavin (30 µg), and iodine (3.4 µg). With minimal fats (0.8 mg) and carbohydrates (6.1 mg), the leaves are light and digestible. Fibre (2.3 mg) and protein (3.1 mg) act synergistically toward maintaining gut health [1]. The constituents of betel leaf are illustrated in Fig. 1, while its various applications are summarized in Fig. 2.

Applications of AI, such as imaging neural networks and feature extraction, enable machine learning and deep learning models for plant leaf disease diagnosis and classification [2]. The classification of betel leaf diseases can be enhanced through imaging deep-learning models, multimodal data fusion, and Agro-meteorological sensor data, improving plant disease diagnosis [3]. The primary dataset of various crops has been collected by researchers in controlled and on-field environments. Further, Artificial Intelligence techniques are applied to the collected dataset to gain insights from it [4,5].

## 3 Data Description

**Fig 3:**
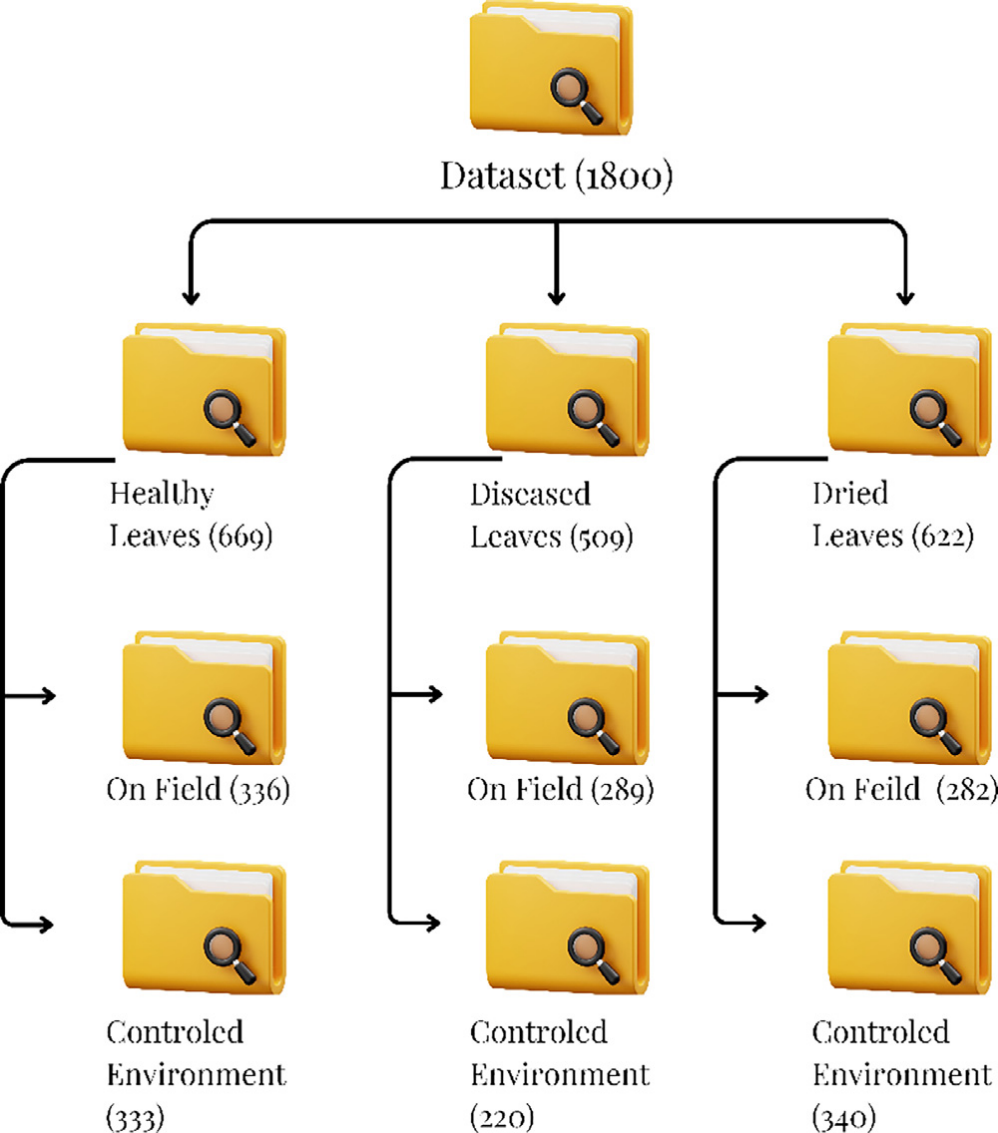
Dataset organization.

The researchers built a dataset with 1,800 high-resolution images (1080 × 1080 pixels) classified into Healthy (669 images), Diseased (509 images), and Dried (622 images) Leaves, for cataloging the different conditions of betel leaves. The images were captured in On-Field and Controlled Environment settings at Veer, Taluka-Purandar, Pune, India. On-Field images were captured under natural farming conditions, with variations in lighting, background and leaf orientation. In contrast, Controlled Environment images were taken indoors with consistent lighting and clean backgrounds to eliminate visual-noise and enhance clarity (Fig. 3).

Specifically, the dataset contains 336 Healthy, 289 Diseased, and 282 Dried Leaves under On-Field conditions, while the figures for leaves under controlled conditions are 333 Healthy, 220 Diseased, and 340 Dried specimens. The above bifurcation of collected images demonstrates a reasonably balanced dataset. In addition, the recent advances in machine learning, including one-shot, few-shot, and zero-shot learning techniques, are interesting potential solutions for addressing class imbalance by allowing a model to learn from only a few examples per class. The proposed work is a novel addition, as prior studies on betel leaves have focused largely on their medicinal and phytochemical aspects rather than image-based classification [6].

Logically, there should be three sets, which are put in their respective folders. Healthy Leaves folder has a total of 669 photos of fresh and intact betel leaves. The Diseased Leaves folder contains photographs of leaves that were infected, discoloured, and otherwise showing associated symptoms that give proof, numbering a total of 509. The Dried Leaves folder has a total of 622 photographs of dried leaves, either naturally or artificially [7].

**Fig 4:**
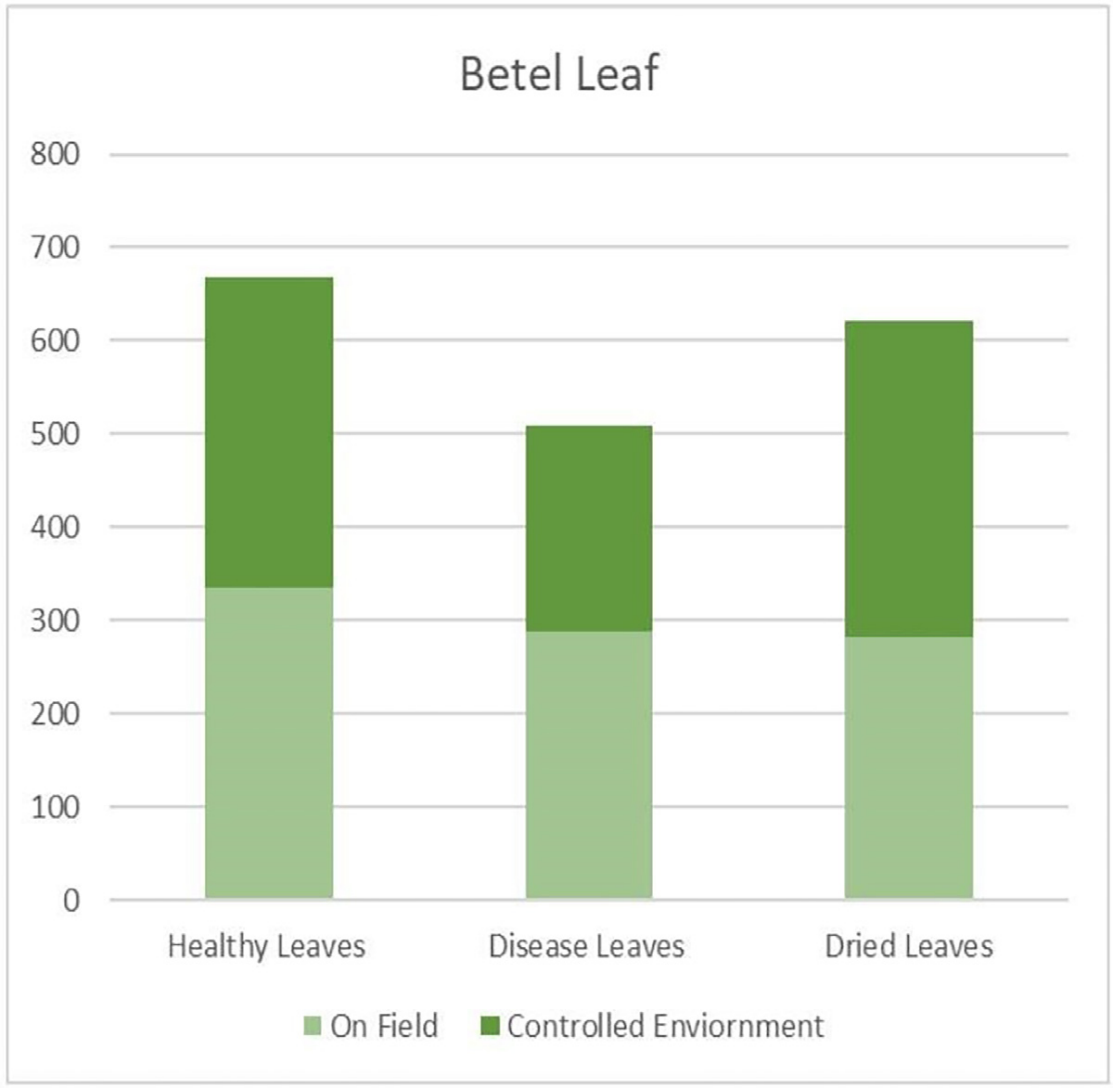
Dataset visualization.

For the purposes of well balanced datasets from exposure to different environmental conditions, the individual categories have subcategories: On-Field and Controlled Environment. The structured image collection enables a thorough representation of betel leaves, making this dataset applicable for research into plant health and quality evaluation (Fig. 4).

## 4 Experimental Design, Materials and Methods

### 4.1 Experimental design

**Fig 5:**
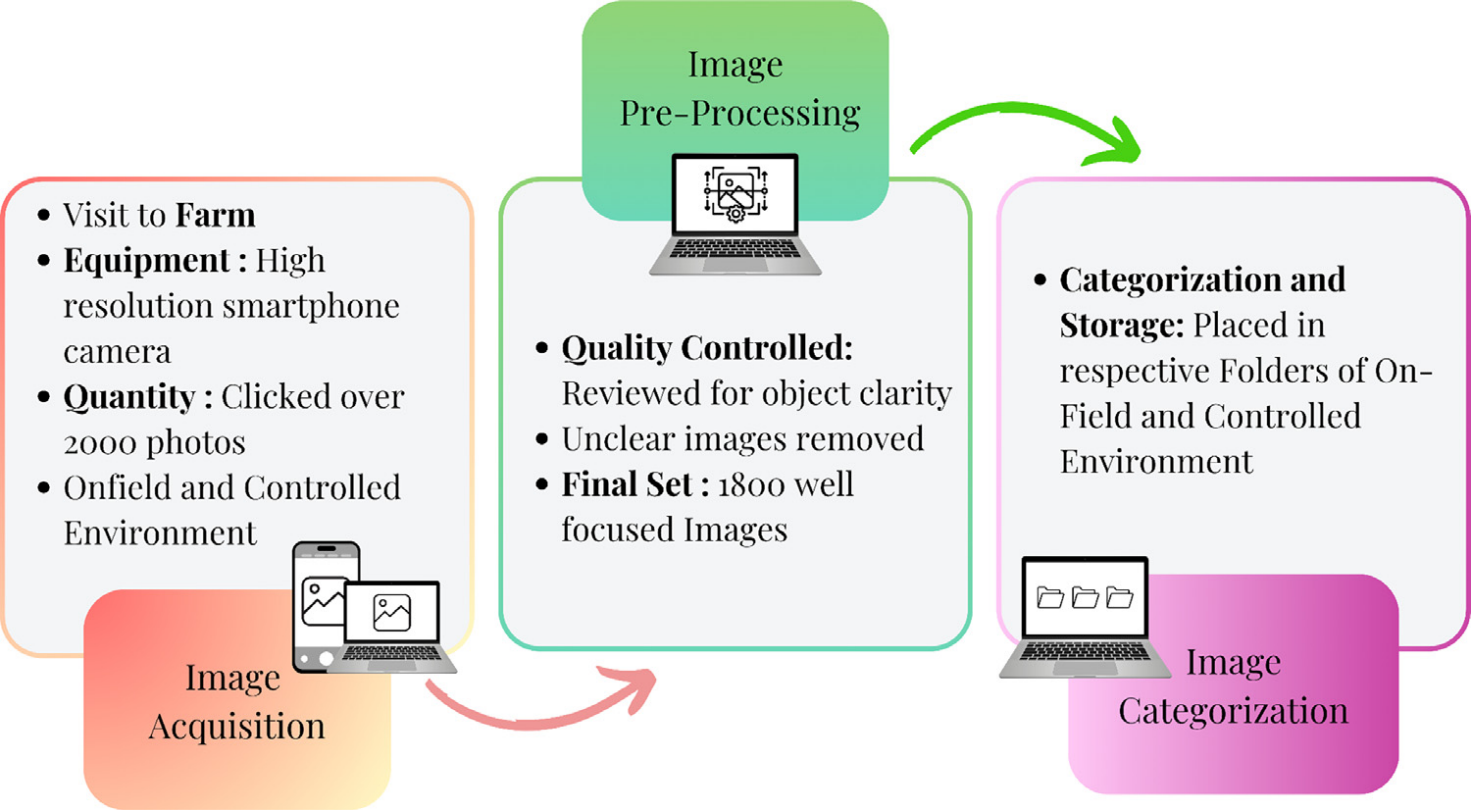
Data acquisition steps.

The Betel Leaf dataset has been created using the high-resolution pro camera of the Samsung Galaxy S23 smartphone in 1:1 ratio, as seen in Fig. 5. The dataset collection process was carried out in three main parts, including field visits and controlled environment observations; thus providing a varied and complete dataset.

Phase 1: Image Acquisition (January)

Collection of daytime samples from the same betel leaf farm for camps with respect to conditions at which the leaves are sometimes kept in controlled conditions for the study of their compare properties sealed under constant conditions. The procedure followed:

• Identifying Healthy (Fresh), Diseased, and Dried Leaves from within the farm. Capturing images of all three leaf categories of Farm: Healthy (Fresh), Diseased, and Dried Leaves.
• Observing leaves in a Controlled Environment.
• Capturing images of all three types in the Controlled Environment: Healthy (Fresh), Diseased, and Dried Leaves.

Phase 2: Image Preprocessing (February)

• In the image preprocessing phase, all captured images were subjected to manual quality and consistency checks. The final dataset included only those images that were clear, well focused, properly illuminated, and featured visible leaf characteristics. Anything that visually deviated from their specifications, such as blurry, overexposed, or underexposed images, or any other poorly framed photos were discarded.
• The images were analysed; the good ones are selected and entered into the dataset,
• The selected images undergo preprocessing steps like cropping, resizing, and enhancement when needed.

Phase 3: Image Categorization (March)

This phase was collecting the images into three specific categories. This was critical to developing a very organized and usable part of the dataset. It entailed two major stages.

1. Image Format and Unique Identification: All images acquired by the authors were in JPEG format to guard the integrity of the dataset from loss or compatibility issues. To facilitate tracking and reference, each image was assigned a unique identifier. Images taken on-field were thus labeled in the format (OF_Healthy_Leaf_no., OF_Diseased_Leaf_no., and OF_Dried_Leaf_no). In contrast, controlled images were tagged according to (CE_Healthy_Leaf_no., CE_Diseased_Leaf_no., and CE_Dried_Leaf_no.). This systematic naming convention enhances clarity and organization in the dataset. Verification of image labels was done by domain experts, comprising well-versed farmers and agricultural specialists. Their expertise became crucial in accurately identifying and confirming the conditions illustrated in the image and thus establishing reliable and credible dataset.
2. Categorization: Each image was meticulously classified into its respective category: healthy, diseased, or dried. Table 1 presents sample images representing each category.

**Table 1:** Sample images of each category.

### 4.2 Materials

This section covers about how the images have been finalized in the Betel Leaf database, along with the details of the camera that had captured them.

Camera Specifications:

• Brand and Model: Samsung Galaxy S23 (Pro Camera)
• Main Camera: 50 MP, f/1.8

All the images acquired were standardized with dimensions of 1080×1080 pixels taken straight from the 1:1 aspect ratio standard imaging setup. No cropping or resizing was performed on the images, thus ensuring similar treatment for all images without distortion or disruption of the aspect ratio and saved in JPEG format to maintain consistent quality of the images across the batch and compatibility throughout the dataset. Most images were taken on sunny days, during the daytime, under relatively consistent outdoor lighting conditions to minimize variability. During image capture, plants were fully grown, and healthy leaves were noticeably predominant compared to dried and diseased ones, indicating favorable soil and cultivation conditions.

## 5 Methods

Field visits to farms producing cultivation of betel leaves were undertaken for gathering the dataset. The authors did study and captured high-resolution images of betel leaves under various conditions with farmers' collaboration. Categorization of the images into sets healthy, affected, and dried has been undertaken to create a well-structured dataset for analysis and research purposes.

A controlled set of conditions was set up to take high-resolution images of betel leaves. Data having structure for analysis and research was ensured because the authors enumerated leaves at different stages.

**Fig 6:**
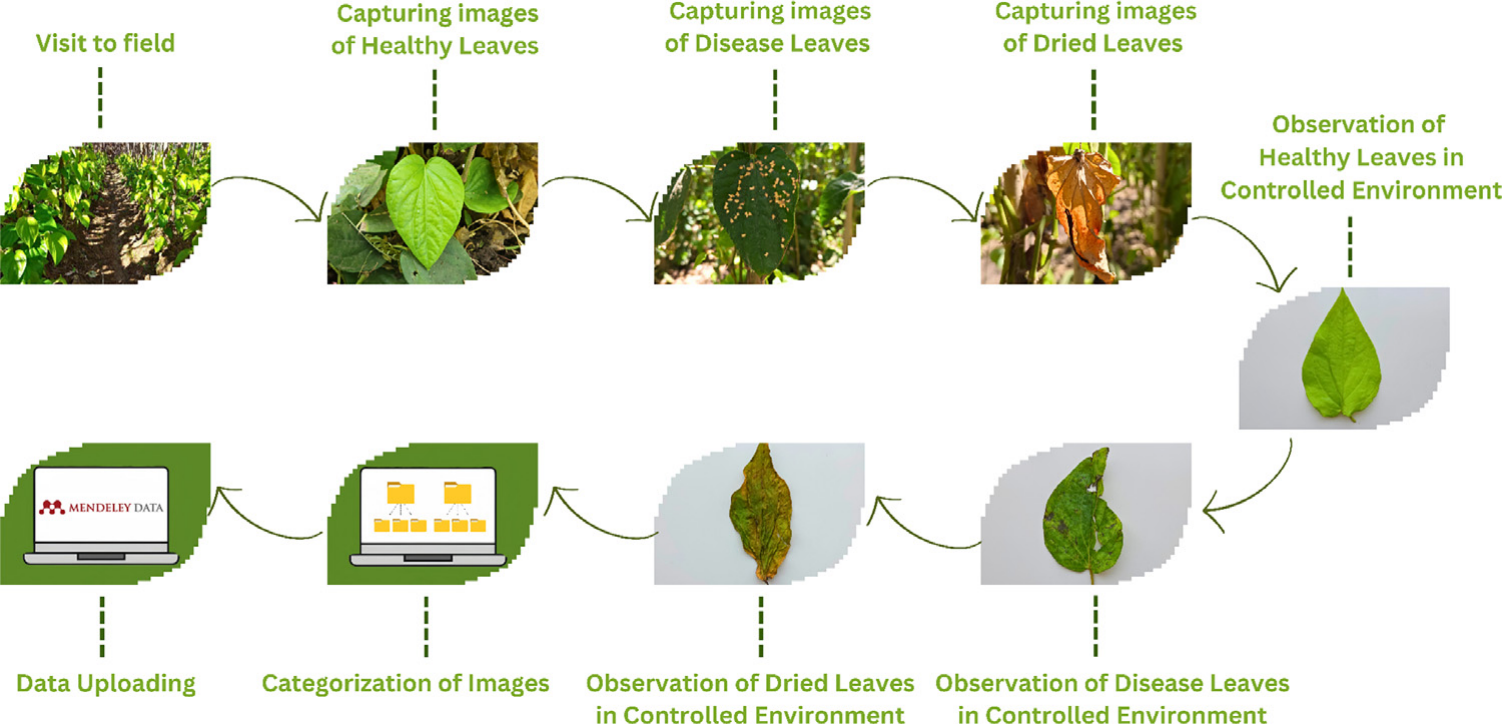
Stage by stage process of dataset creation.

The Betel Leaf dataset was collated from a farm in Veer, Taluka-Purandar, District-Pune, India. The detailed procedure for developing a betel leaf dataset is shown in Fig. 3 (Fig. 6).

### 4.1 Comparison with existing datasets available in literature

Although betel leaf is much appreciated for its cultural, economic, and medicinal values, there is not an extensive image dataset available for AI-based automated classification and health assessment. There are only a handful of datasets, which are very much limited and lack variability in leaf conditions and environmental contexts.

According to Table 1, the Betel Leaf Image Dataset from Bangladesh includes 1,000 images taken only on-field conditions and involves healthy and diseased leaves while excluding completely dried leaves. It also does not have images from a controlled environment. Likewise, the Comprehensive Betel Leaf Disease Dataset for Advanced Pathology Research has more images (2,037), but it covers only dried leaves under controlled conditions and does not provide full category coverage across both the environments (Table 2).

**Table 2:** - Comparative table for Indian betel leaf dataset.

Sr.No.	Dataset Ref. No.	Repository	Total Images	On-Field			Controlled Environment		
				Healthy leaves	Diseased Leaves	Dried Leaves	Healthy Leaves	Diseased Leaves	Dried Leaves
1.	[8]	Betel Leaf image dataset from Bangladesh (Original Images)	1000						
2.	[9]	Comprehensive Betel Leaf Disease Dataset For Advanced Pathology Research (Original_Dataset)	10185						
3.	[7]	Betel Leaf Dataset: A Primary Dataset From Field And Controlled Environment (Authors Dataset)	1800						

In contrast, our dataset fills this crucial gap. It is the only dataset with 1,800 high-resolution images showing all three leaf conditions—Healthy, Diseased, and Dried—recorded with both On-Field and Controlled Environmental settings. This entire structuring allows for higher diversity, realism, and usefulness in training robust machine learning and deep learning models.

## Limitations

This Betel Leaf dataset, mainly contains Healthy (Fresh), Dried, and Diseased Leaves collected from the farm of Pune, Maharashtra, India. However, this dataset is not categorically specific per disease, restricting its use for studies that require determinate identification of various betel leaf diseases.

The dataset for the present research comprises betel leaves that were collected using digital imaging techniques in Veer, Taluka - Purandar, Maharashtra, India (approximate coordinates: Latitude :18.1507784, Longitude :74.0872852). The specific climate and the nature of the soil in this region, along with the agricultural activities, would determine the possible changes on the features of betel leaves such as their size, color tone, and patterns of manifestation of diseases. Models built on this dataset should perform well with data from the same climatic environment but will lag behind when performing with betel leaves from entirely different geographical or climatic regions. Future work should, therefore, consider the expansion of the dataset to also include betel leaves from diverse locations in order to strengthen the robustness and generalizability of the trained models.

## Ethics Statement

The authors confirm that they have read and follow the ethical requirements for publication in Data in Brief and confirm that the current work does not involve human subjects, animal experiments, or any data collected from social media platforms.

## CRediT Author Statement

Gauri Mane: Data Curation, Writing – Review & Editing, Methodology. Raghav Bhise: Data Curation, Writing – Review & Editing, Methodology. Rutuja Kadam: Supervision, Conceptualization. Gagandeep Kaur: Conceptualization, Validation. Devika Verma: Reviewing. Rupali Chopade: Reviewing. Gitanjali Shinde: Supervision, Ghanshyam G. Tejani: Reviewing, Editing, Seyed Jalaleddin Mousavirad: Reviewing, Editing

## Data Availability

Mendeley DataBetel Leaf Dataset: A Primary Dataset From Field And Controlled Environment (Original data).
